# 4,4′-Dibromo-2-nitro­biphen­yl

**DOI:** 10.1107/S1600536812000347

**Published:** 2012-01-11

**Authors:** J. Josephine Novina, G. Vasuki, Sushil Kumar, K. R. Justin Thomas

**Affiliations:** a Department of Physics, Idhaya College for Women, Kumbakonam-1, India; b Department of Physics, Kunthavai Naachiar Govt. Arts College (W) (Autonomous), Thanjavur-7, India; c Organic Materials Lab, Department of Chemistry, Indian Institute of Technology Roorkee, Roorkee 247 667, India

## Abstract

The title compound, C_12_H_7_Br_2_NO_2_, a biphenyl derivative, displays a twisted conformation with the two benzene rings making a dihedral angle of 55.34 (14)°. The dihedral angle between the nitro group and its parent benzene ring is 26.8 (2)°. The crystal structure is stabilized by inter­molecular C—H⋯Br and C—H⋯O inter­actions, which lead to the formation of chains propagating along the *c*-axis direction.

## Related literature

For the use of dibromo-2-nitro-biphenyl as a crucial precursor in the formation of 2,7-disubstituted carbazole derivatives, see: Dierschke *et al.* (2003 ▶); Blouin *et al.* (2007 ▶). For details concerning 3,6-disubstituted analogs, see: Thomas *et al.* (2001 ▶). For related structures, see: Akhter *et al.* (2009 ▶); Hou *et al.* (2011 ▶); Kia *et al.* (2009 ▶); Rajnikant *et al.* (1995 ▶); Sim (1986 ▶).
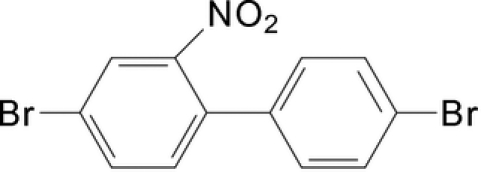


## Experimental

### 

#### Crystal data

C_12_H_7_Br_2_NO_2_
*M* *_r_* = 357.01
Orthorhombic, 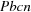

*a* = 15.8761 (14) Å
*b* = 7.4350 (7) Å
*c* = 20.7517 (13) Å
*V* = 2449.5 (4) Å^3^
*Z* = 8
Mo *K*α radiation
μ = 6.61 mm^−1^
*T* = 293 K
0.40 × 0.35 × 0.30 mm

#### Data collection

Bruker Kappa APEXII CCD diffractometer
Absorption correction: multi-scan (*SADABS*; Bruker, 2004 ▶) *T*_min_ = 0.089, *T*_max_ = 0.138
13009 measured reflections
2607 independent reflections
1521 reflections with *I* > 2σ(*I*)
*R*_int_ = 0.045

#### Refinement

*R*[*F* ^2^ > 2σ(*F* ^2^)] = 0.042
*wR*(*F* ^2^) = 0.095
*S* = 1.00
2607 reflections
154 parameters
H-atom parameters constrained
Δρ_max_ = 0.40 e Å^−3^
Δρ_min_ = −0.70 e Å^−3^

### 

Data collection: *APEX2* (Bruker, 2004 ▶); cell refinement: *APEX2* and *SAINT* (Bruker, 2004 ▶); data reduction: *SAINT* and *XPREP* (Bruker, 2004 ▶); program(s) used to solve structure: *SHELXS97* (Sheldrick, 2008 ▶); program(s) used to refine structure: *SHELXL97* (Sheldrick, 2008 ▶); molecular graphics: *ORTEP-3* (Farrugia, 1997 ▶); software used to prepare material for publication: *PLATON* (Spek, 2009 ▶).

## Supplementary Material

Crystal structure: contains datablock(s) I, global. DOI: 10.1107/S1600536812000347/su2358sup1.cif

Structure factors: contains datablock(s) I. DOI: 10.1107/S1600536812000347/su2358Isup2.hkl

Supplementary material file. DOI: 10.1107/S1600536812000347/su2358Isup3.cml

Additional supplementary materials:  crystallographic information; 3D view; checkCIF report

## Figures and Tables

**Table 1:** Hydrogen-bond geometry (Å, °)

*D*—H⋯*A*	*D*—H	H⋯*A*	*D*⋯*A*	*D*—H⋯*A*
C2—H2⋯Br2^i^	0.93	2.89	3.798 (3)	165
C9—H9⋯O2^ii^	0.93	2.57	3.454 (5)	159

Symmetry codes: (i) 
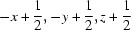
; (ii) 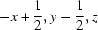.

**Fig. 1.**
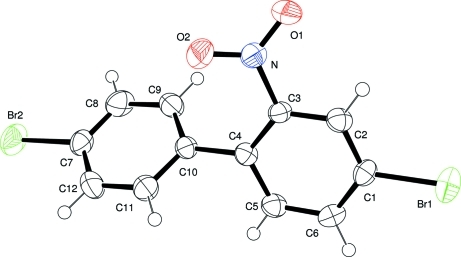
The molecular structure of the title compound, with atom numbering and displacement ellipsoids drawn at the 50% probability level.

**Fig. 2.**
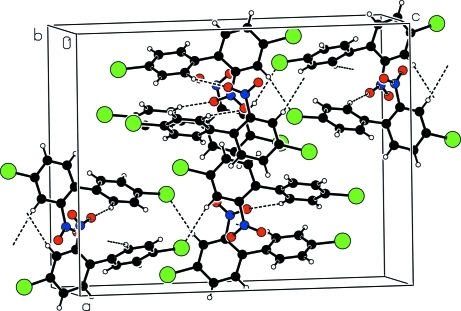
Crystal packing of the title compound viewed along the b axis, showing the C-H···Br and C-H···O interactions as dashed lines (see Table 1 for details).

## supplementary crystallographic information

### Comment

Biphenyl and its derivatives are important industrial intermediates used in the production of heat transfer fluids, formulations for dye carriers used in textile dyeing and polychlorinated biphenyls used in insecticides. The C—Br bond in certain biphenyl derivatives is labile and the compound can be used for the preparation of carboxylic acid functionalized biphenyl derivatives. 4,4'-Dibromo-2-nitro-biphenyl is used as an crucial precursor in the formation of 2,7-disubstituted carbazole derivatives (Dierschke *et al.*, 2003; Blouin *et al.*, 2007), which have been found to display unusual electronic properties when compared to the 3,6-disubstituted analogs (Thomas *et al.*, 2001).

Structures of biphenyl and its derivatives have been studied extensively in the past and even now, because of the differences found in the inter–ring torsion angle φ in the solid state (Rajnikant *et al.*, 1995), which alters the electronic properties. In a continuation of our on-going research program aimed at investigating the trends in crystallization and crystal growth of some substituted biphenyl derivatives, the crystal and molecular structure of the title compound is presented herein.

The title compound (Fig. 1) displays a twisted conformation with the two benzene rings making a dihedral angle of 55.34 (14)°. The dihedral angle between the nitro group and its parent benzene ring is 26.76 (20)°. The length of the bond connecting the phenyl rings, 1.483 (5) Å, is close to the standard value of 1.48 Å for a C*sp*^2^—C*sp*^2^ single bond, and to that observed in similar structures, for example 2-Bromo-4'-phenylacetophenone (II) [Sim, 1986], 4-Methoxy-2-nitro-4'-(trifluoromethyl)-biphenyl (III) [Hou *et al.*, 2011], and *N*-[1-(Biphenyl-4-yl)ethylidene]-*N*'-(2,4-dinitrophenyl)hydrazine (IV) [Kia *et al.*, 2009]. All the bond lengths and angles are comparable to those obserbed in related structures. The distribution of bond angles around atom C4 is quite similar to that reported for 2-substituted biphenyls with angle C3—C4—C5 considerably less than 120° and angle C3—C4—C10 greater than 120°, as observed in the related structures, Biphenyl-2-methanol (V) [Rajnikant *et al.*, 1995], and 4-(4-Nitrophenoxy) biphenyl (VI) [Akhter *et al.*, 2009]. The two bromine atoms and the nitro group are in antiperiplanar positions with respect to the benzene rings to which they are attached.

In the crystal, there are no classical hydrogen bonds and the crystal structure is stabilized by intermolecular C—H···Br and C—H···O interactions (Table 1 and Fig. 2), which lead to the formation of one-dimensional chains propagating along the c axis direction.

### Experimental

The title compound was synthesized by following a protocol reported in literature (Dierschke *et al.*, 2003), in which the expensive fuming nitric acid was replaced by a potassium nitrate and sulfuric acid mixture. 4,4,-Dibromobiphenyl (25 g) was suspended in 120 ml of glacial acetic acid and heated to 363 K for 45 min. with efficient stirring. A preformed mixture of KNO_3_ (18 g) and H_2_SO_4_ (36 ml) was added drop wise maintaining the temperature at 363 K. After the addition was complete the mixture was heated and stirred for further 30 min. On completion of the reaction, the mixture was cooled and poured into water. The yellow precipitate formed was filtered and recrystallized from ethanol [Yield: 82%]. The spectral data matched with those reported in the literature (Dierschke *et al.*, 2003).

### Refinement

All the H atoms were included in calculated positions and treated as riding atoms: C–H = 0.93 Å with *U*_iso_(H) = 1.2*U*_eq_(C).

### Crystal data

**Table:**

C_12_H_7_Br_2_NO_2_	*F*(000) = 1376
*M**_r_* = 357.01	*D*_x_ = 1.936 Mg m^−^^3^
Orthorhombic, *P**b**c**n*	Mo *K*α radiation, λ = 0.71073 Å
Hall symbol: -P 2n 2ab	Cell parameters from 25 reflections
*a* = 15.8761 (14) Å	θ = 20–30°
*b* = 7.4350 (7) Å	µ = 6.61 mm^−^^1^
*c* = 20.7517 (13) Å	*T* = 293 K
*V* = 2449.5 (4) Å^3^	Needle, yellow
*Z* = 8	0.40 × 0.35 × 0.30 mm

### Data collection

**Table:**

Bruker Kappa APEXII CCD diffractometer	2607 independent reflections
Radiation source: fine-focus sealed tube	1521 reflections with *I* > 2σ(*I*)
graphite	*R*_int_ = 0.045
ω and φ scan	θ_max_ = 27.0°, θ_min_ = 2.3°
Absorption correction: multi-scan (*SADABS*; Bruker, 2004)	*h* = −18→20
*T*_min_ = 0.089, *T*_max_ = 0.138	*k* = −9→9
13009 measured reflections	*l* = −16→26

### Refinement

**Table:**

Refinement on *F*^2^	Primary atom site location: structure-invariant direct methods
Least-squares matrix: full	Secondary atom site location: difference Fourier map
*R*[*F*^2^ > 2σ(*F*^2^)] = 0.042	Hydrogen site location: inferred from neighbouring sites
*wR*(*F*^2^) = 0.095	H-atom parameters constrained
*S* = 1.00	*w* = 1/[σ^2^(*F*_o_^2^) + (0.041*P*)^2^ + 1.6705*P*] where *P* = (*F*_o_^2^ + 2*F*_c_^2^)/3
2607 reflections	(Δ/σ)_max_ = 0.001
154 parameters	Δρ_max_ = 0.40 e Å^−^^3^
0 restraints	Δρ_min_ = −0.70 e Å^−^^3^

### Special details

**Table:**

Geometry. All e.s.d.'s (except the e.s.d. in the dihedral angle between two l.s. planes) are estimated using the full covariance matrix. The cell e.s.d.'s are taken into account individually in the estimation of e.s.d.'s in distances, angles and torsion angles; correlations between e.s.d.'s in cell parameters are only used when they are defined by crystal symmetry. An approximate (isotropic) treatment of cell e.s.d.'s is used for estimating e.s.d.'s involving l.s. planes.
Refinement. Refinement of *F*^2^ against ALL reflections. The weighted *R*-factor *wR* and goodness of fit *S* are based on *F*^2^, conventional *R*-factors *R* are based on *F*, with *F* set to zero for negative *F*^2^. The threshold expression of *F*^2^ > σ(*F*^2^) is used only for calculating *R*-factors(gt) *etc*. and is not relevant to the choice of reflections for refinement. *R*-factors based on *F*^2^ are statistically about twice as large as those based on *F*, and *R*- factors based on ALL data will be even larger.

### Fractional atomic coordinates and isotropic or equivalent isotropic displacement parameters (Å^2^)

**Table:**

	*x*	*y*	*z*	*U*_iso_*/*U*_eq_	
Br1	0.03710 (3)	0.09619 (7)	0.65877 (2)	0.05528 (18)	
Br2	0.15585 (3)	0.40559 (8)	0.15916 (2)	0.06048 (19)	
C7	0.1445 (2)	0.3592 (6)	0.24826 (17)	0.0382 (10)	
C9	0.1668 (2)	0.1683 (6)	0.33822 (18)	0.0410 (10)	
H9	0.1880	0.0618	0.3552	0.049*	
C1	0.0668 (2)	0.1609 (5)	0.57419 (16)	0.0321 (9)	
O1	0.30721 (17)	0.1125 (4)	0.51041 (14)	0.0524 (8)	
C11	0.0974 (3)	0.4456 (6)	0.35168 (19)	0.0438 (11)	
H11	0.0702	0.5287	0.3779	0.053*	
N	0.26111 (19)	0.2075 (5)	0.47864 (15)	0.0342 (8)	
C5	0.0275 (2)	0.2556 (5)	0.46893 (18)	0.0343 (10)	
H5	−0.0144	0.2906	0.4403	0.041*	
O2	0.28561 (16)	0.3083 (5)	0.43688 (14)	0.0518 (8)	
C4	0.1106 (2)	0.2495 (5)	0.44718 (16)	0.0289 (9)	
C2	0.1493 (2)	0.1555 (5)	0.55524 (17)	0.0329 (9)	
H2	0.1910	0.1212	0.5842	0.039*	
C10	0.1272 (2)	0.2897 (5)	0.37828 (16)	0.0292 (9)	
C6	0.0050 (2)	0.2116 (5)	0.53120 (17)	0.0362 (10)	
H6	−0.0511	0.2162	0.5440	0.043*	
C8	0.1752 (2)	0.2031 (7)	0.27314 (19)	0.0467 (12)	
H8	0.2018	0.1201	0.2465	0.056*	
C3	0.1701 (2)	0.2011 (5)	0.49318 (17)	0.0280 (9)	
C12	0.1067 (3)	0.4826 (6)	0.28703 (19)	0.0476 (11)	
H12	0.0873	0.5908	0.2701	0.057*	

### Atomic displacement parameters (Å^2^)

**Table:**

	*U*^11^	*U*^22^	*U*^33^	*U*^12^	*U*^13^	*U*^23^
Br1	0.0631 (3)	0.0675 (4)	0.0352 (3)	−0.0030 (3)	0.01363 (19)	0.0059 (2)
Br2	0.0541 (3)	0.0981 (5)	0.0293 (2)	0.0006 (3)	0.00445 (18)	0.0110 (3)
C7	0.034 (2)	0.056 (3)	0.025 (2)	−0.002 (2)	−0.0014 (17)	0.002 (2)
C9	0.046 (3)	0.042 (3)	0.034 (2)	0.011 (2)	0.0008 (17)	0.003 (2)
C1	0.035 (2)	0.036 (3)	0.0254 (19)	−0.0034 (18)	0.0028 (16)	−0.0055 (18)
O1	0.0363 (16)	0.074 (2)	0.0470 (19)	0.0164 (17)	−0.0024 (13)	0.0104 (17)
C11	0.054 (3)	0.045 (3)	0.033 (2)	0.015 (2)	0.0106 (18)	0.001 (2)
N	0.0308 (18)	0.042 (2)	0.0297 (18)	−0.0003 (17)	−0.0018 (14)	−0.0033 (17)
C5	0.032 (2)	0.039 (3)	0.031 (2)	0.0032 (19)	−0.0054 (15)	−0.0030 (19)
O2	0.0366 (18)	0.066 (2)	0.0530 (19)	−0.0062 (15)	0.0047 (13)	0.0157 (18)
C4	0.031 (2)	0.029 (2)	0.027 (2)	0.0010 (17)	−0.0027 (15)	−0.0020 (17)
C2	0.031 (2)	0.037 (3)	0.030 (2)	0.0020 (18)	−0.0052 (16)	−0.0032 (18)
C10	0.029 (2)	0.035 (3)	0.0239 (18)	0.0014 (18)	−0.0032 (15)	−0.0006 (19)
C6	0.028 (2)	0.040 (3)	0.040 (2)	0.0018 (19)	0.0046 (17)	−0.005 (2)
C8	0.049 (3)	0.059 (3)	0.032 (2)	0.010 (2)	0.0054 (17)	−0.012 (2)
C3	0.024 (2)	0.032 (2)	0.029 (2)	0.0016 (17)	0.0005 (13)	−0.0016 (18)
C12	0.057 (3)	0.049 (3)	0.037 (2)	0.012 (2)	0.0012 (19)	0.013 (2)

### Geometric parameters (Å, °)

**Table:**

Br1—C1	1.880 (3)	N—O2	1.210 (4)
Br2—C7	1.890 (4)	N—C3	1.477 (4)
C7—C8	1.361 (6)	C5—C6	1.380 (5)
C7—C12	1.361 (6)	C5—C4	1.395 (5)
C9—C10	1.378 (5)	C5—H5	0.9300
C9—C8	1.382 (5)	C4—C3	1.390 (5)
C9—H9	0.9300	C4—C10	1.484 (5)
C1—C2	1.368 (5)	C2—C3	1.372 (5)
C1—C6	1.380 (5)	C2—H2	0.9300
O1—N	1.212 (4)	C6—H6	0.9300
C11—C10	1.369 (5)	C8—H8	0.9300
C11—C12	1.377 (5)	C12—H12	0.9300
C11—H11	0.9300		
			
C8—C7—C12	120.6 (4)	C5—C4—C10	118.2 (3)
C8—C7—Br2	119.5 (3)	C1—C2—C3	119.5 (3)
C12—C7—Br2	119.8 (3)	C1—C2—H2	120.2
C10—C9—C8	120.7 (4)	C3—C2—H2	120.2
C10—C9—H9	119.6	C11—C10—C9	118.0 (3)
C8—C9—H9	119.6	C11—C10—C4	119.8 (3)
C2—C1—C6	120.3 (3)	C9—C10—C4	122.0 (4)
C2—C1—Br1	120.1 (3)	C1—C6—C5	119.0 (3)
C6—C1—Br1	119.7 (3)	C1—C6—H6	120.5
C10—C11—C12	121.7 (4)	C5—C6—H6	120.5
C10—C11—H11	119.2	C7—C8—C9	119.7 (4)
C12—C11—H11	119.2	C7—C8—H8	120.1
O2—N—O1	123.8 (3)	C9—C8—H8	120.1
O2—N—C3	118.7 (3)	C2—C3—C4	123.1 (3)
O1—N—C3	117.5 (3)	C2—C3—N	115.8 (3)
C6—C5—C4	122.7 (3)	C4—C3—N	121.1 (3)
C6—C5—H5	118.6	C7—C12—C11	119.2 (4)
C4—C5—H5	118.6	C7—C12—H12	120.4
C3—C4—C5	115.4 (3)	C11—C12—H12	120.4
C3—C4—C10	126.4 (3)		

### Hydrogen-bond geometry (Å, °)

**Table:**

*D*—H···*A*	*D*—H	H···*A*	*D*···*A*	*D*—H···*A*
C2—H2···Br2^i^	0.93	2.89	3.798 (3)	165
C9—H9···O2^ii^	0.93	2.57	3.454 (5)	159

Symmetry codes: (i) −*x*+1/2, −*y*+1/2, *z*+1/2; (ii) −*x*+1/2, *y*−1/2, *z*.
